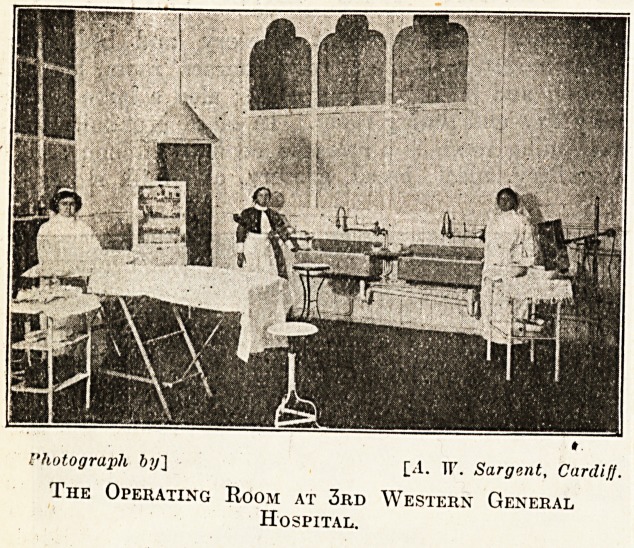# The Making of a Military Hospital

**Published:** 1914-11-14

**Authors:** 


### THE MAKING OF A MILITARY HOSPITAL.

### How the Transformation at Cardiff was Effected.

The five transformed schools comprising the 3rd Western General Hospital, Cardiff, are striking testimony to the skill and energy displayed by Colonel Bruce Vaughan, V.D., J.P., who, as a military member of the Glamorgan Territorial Association, was appointed honorary architect by that body for the work under review. As the public know, Colonel Vaughan's name has become inseparably associated with that of King Edward VII.'s Hospital, Cardiff, by reason of the work accomplished on its behalf in every direction through which its leading position has been built up. It was on August 10 that, as a result of the influence exerted by Major-General Lee (chairman of the board of management of this hospital) and Colonel Vaughan, 150 beds were placed at the disposal of the War Office for wounded soldiers in an institution fitted in every way to give treatment of the best and most scientific character. The five schools were old and out of date, had no supply of hot water, no electric light or other fittings, and no proper heating apparatus. To convert them into a military hospital accommodating several hundred patients, and have the beds ready for occupation in a month, was no small task, but it was accomplished successfully.

Mr. Lloyd George visited the 3rd Western General Hospital on September 29, and in the course of conversation said: "I am delighted with the arrangements. It is really a very great triumph the way in which the schools have been converted into hospitals for the treatment of wounded soldiers. I congratulate Cardiff upon the resource it has displayed."

The three hospitals are centrally situated and at a convenient distance from the Nurses' Home and the barracks. The following is a description of one of the buildings, and may be taken as typical of the whole: On the ground floor near the main entrance is the telephone room. There are fourteen wards on this floor with excellent lavatory and bed-pan accommodation, doctors' room, nurses' room, bathrooms, and ladies' sewing room; also a large and lofty day and dining room. In close proximity to the dining room is a splendid cooking kitchen and a scullery, meat, bread, grocery, and milk stores,

*Photograph by*] [*A. W. Sargent, Cardiff.*
A Ward in 3rd Western General Hospital.

with bins for flour, rice, tapioca, sugar, etc., and a serving counter. The covered playground has been well planned and converted into a *post-mortem* room, viewing room, and mortuary. There are commodious linen, pack, and mufti stores, with special boxes for private kit. In addition there are two large stores and a covered space for an incinerator. The outer playgrounds are large and of cheerful aspect, and make excellent recreation places for those convalescing. On the first floor are eight wards with lavatory accommodation as on the ground floor, and excellent rooms for nurses and for the male staff. There is an operating theatre, sterilising and anæsthetising and

*Photograph by*] [*A. W. Sargent, Cardiff.*
Arrival of Wounded at 3rd Western General Hospital.

dressing rooms, *x*-ray department, and laboratory. There are also a suite of offices approached by a private staircase, and comprising the commanding officer's and the registrar's rooms, and a room for the clerical staff. In the corridors are stands for sterilisers and cupboards for a twenty-four hours' supply of linen. Ward kitchens are found on each floor.

*Photograph by*] [*A. W. Sargent, Cardiff.*
THE OPERATING ROOM AT 3RD WESTERN GENERAL HOSPITAL.

The heating, lighting, ventilation, and the hot and cold water supply in these hospitals are good, and all the buildings are fitted with telephones and speaking-tubes. Fire-extinguishers and buckets are provided in the most convenient positions.

The whole of the hospitals have been made hygienically complete, the floors have been treated with "Solignum," and the walls with coloured "Wal-per-mer."

The names of the schools now forming the 3rd Western General Hospital at Cardiff, with their present use and accommodation, are:

Albany Road Schools, converted into a surgical hospital, containing 156 beds; Splott Road Schools, converted into a surgical hospital, containing 145 beds; Howard Gardens Secondary Schools, converted into a medical hospital, containing 155 beds; Pupil Teachers' Centre, converted into a nurses' home, accommodating seventy nurses; Boys' Intermediate School, Newport Road, converted into barracks for the Red Cross and St. John Ambulance Societies.

The contractors were: Messrs. Proger and Sons (plumbing), Messrs. Cross Bros. (plumbing), Messrs. Knox and Wells (carpentry and joinery), Messrs. Jenks and Co. (painting), and Messrs. Herbert Lewis and Fletcher (electric fittings), all of Cardiff.

## Figures and Tables

**Figure f1:**
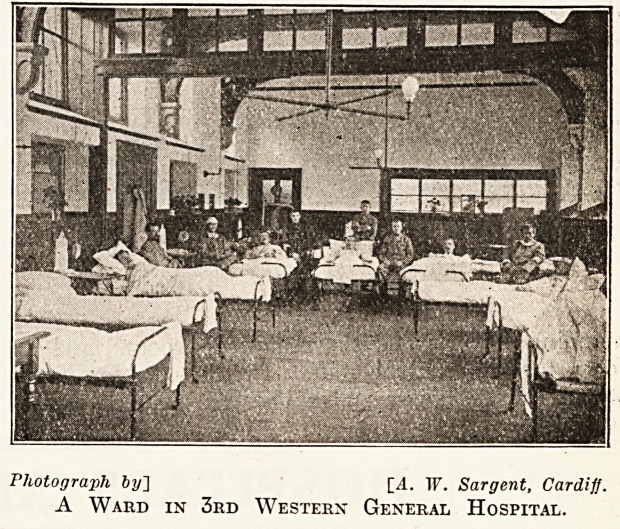


**Figure f2:**
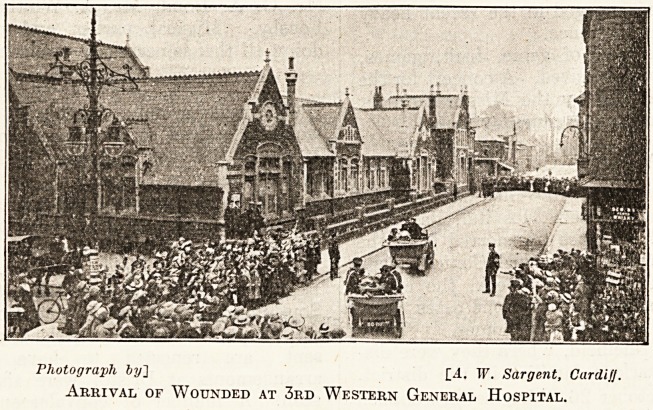


**Figure f3:**